# Antioxidative and Analgesic Effects of Naringin through Selective Inhibition of Transient Receptor Potential Vanilloid Member 1

**DOI:** 10.3390/antiox11010064

**Published:** 2021-12-28

**Authors:** Sanung Eom, Bo-Bae Lee, Shinhui Lee, Youngseo Park, Hye Duck Yeom, Tae-Hwan Kim, Seung-Hee Nam, Junho H. Lee

**Affiliations:** 1 Department of Biotechnology, Chonnam National University, Gwangju 61186, Korea; yeomself2355@gmail.com (S.E.); dltlstn39@gmail.com (S.L.); ppyoungs28@gmail.com (Y.P.); hyeduck@gmail.com (H.D.Y.); 2 Fruit Research Institute of Jeollanamdo Agricultural Research and Extension Services, Haenam, Naju 59021, Korea; lbb0509@korea.kr; 3 Department of Animal Science, Chonnam National University, Gwangju 61186, Korea; grassl@chonnam.ac.kr; 4 Institute of Agricultural Science and Technology, Chonnam National University, Gwangju 61186, Korea

**Keywords:** naringin, antioxidant, TRPV1, capsaicine-selective inhibition, analgesic, free reactive oxygen species

## Abstract

Transient receptor potential vanilloid member 1 (TRPV1) is activated in response to capsaicin, protons, temperature, and free reactive oxygen species (ROS) released from inflammatory molecules after exposure to harmful stimuli. The expression level of TRPV1 is elevated in the dorsal root ganglion, and its activation through capsaicin and ROS mediates neuropathic pain in mice. Its expression is high in peripheral and central nervous systems. Although pain is a response evolved for survival, many studies have been conducted to develop analgesics, but no clear results have been reported. Here, we found that naringin selectively inhibited capsaicin-stimulated inward currents in *Xenopus* oocytes using a two-electrode voltage clamp. The results of this study showed that naringin has an IC_50_ value of 33.3 μM on TRPV1. The amino acid residues D471 and N628 of TRPV1 were involved in its binding to naringin. Our study bridged the gap between the pain suppression effect of TRPV1 and the preventive effect of naringin on neuropathic pain and oxidation. Naringin had the same characteristics as a model selective antagonist, which is claimed to be ideal for the development of analgesics targeting TRPV1. Thus, this study suggests the applicability of naringin as a novel analgesic candidate through antioxidative and analgesic effects of naringin.

## 1. Introduction

**Figure 1:**
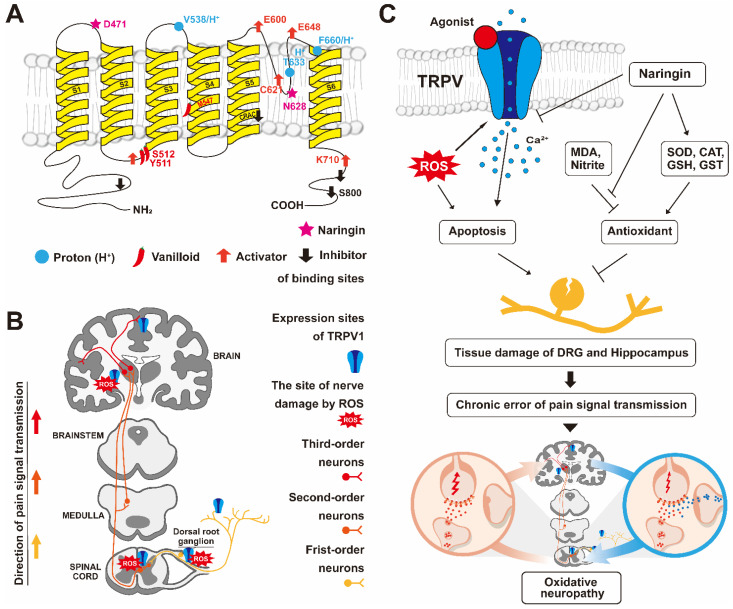
Schematic diagram of the various interaction sites of TRPV1 and the mechanism underlying the neuroprotective effect of naringin in the pain pathway: (**A**) one subunit of TRPV1 has six transmembrane domains (yellow) and a pore loop (S5–S6). TRPV1 can function only when these four subunits form a tetramer. The light blue circle is the location where the proton binds to TRPV1. The pepper-shaped symbol is the site where vanilloids such as capsaicin bind. The star shape is the binding site of naringin found in our present study. (**B**) The pain transmission pathway. The signal transduction process of pain involves three stages. Electrical signals are transmitted from the first-order neurons (yellow neurons) to the third-order neurons (red neurons), and finally, the brain perceives the pain. The blue receptor symbol is TRPV1 and marks the tissue location where it is expressed. (**C**) Naringin has a cytoprotective effect mediated through inhibition of neuronal hyperexcitation and oxidative stress via TRPV. The death of neuronal cells remains a permanent error in the signaling system, resulting in neuropathic pain. GSH is glutathione, MDA is malondialdehyde, SOD is superoxide dismutase, CAT is catalase, and GST is glutathione-S-transferase.

Many neurodegenerative dysfunctions are associated with excessive production of free reactive oxygen species (ROS), which may be eliminated or inhibited by antioxidants [1]. Herbal plants, including several fruits and vegetables, are compounds of natural antioxidants such as phenolic compounds and flavonoids, which exert antioxidant properties [2]. Pain is a complex phenomenon attributed to the transmission of electrical signals through neurons to the brain [3,4]. It is a response evolved to protect our body from external and internal dangers but also reduces our quality of life. Pain can be classified in various types but typically is categorized as neuropathic or nociceptive type [5]. Neuropathic pain is associated with the malfunction of the neuronal system in response to injury, disease, or inflammation and serves as a message to the brain [6,7]. Neuropathic pain is chronic when nociceptors interact with internal and external harmful stimuli, resulting in the generation of electrical signals in afferent sensory neurons. Nociceptive pain arises from the transmission of these electrical signals to the brain through sensory nerve fibers [8,9]. In general, as shown in Figure 1, pain is an electrical signal (message of pain) that is transmitted through first-order neurons (peripheral nerve bundles), second-order neurons (spinal nerve bundles), and third-order neurons (cranial nerves) [10,11,12].

Thirty members of the transient receptor potential (TRP) superfamily of proteins act as sensors for heat, cold, osmosis, volume, stretch, tremor, and taste [13,14]. Among them, transient receptor potential vanilloid member 1 (TRPV1) is a nonselective cation channel expressed in the peripheral and central nervous systems [15]. The activation of TRPV1 in response to stimuli such as protons, heat, and capsaicin is the first step in the pain mechanism that produces electrical signals as the message [16,17]. Studies have been conducted on the relationship between pain and TRPV1 function by investigating the interaction between TRPV1 and capsaicin of vanilloids [3,16,18,19,20,21]. TRPV1 was found to be involved in nociceptive pain as well as chronic pain of inflammatory and peripheral neuropathy.

The challenge in developing analgesics based on various pain mechanisms is to target the immune system, calcium channels, neurokinin 1 (NK1) receptors, and gamma-aminobutyric acid (GABA) receptors and remains largely unaddressed [11]. No effective analgesic have been found to date, and the development of any new analgesics targeting TRPV1 [21,22,23,24] to selectively inhibit only capsaicin activity without affecting the proton activity is imperative [19,25,26].

Naringin, one of the main components of citrus fruits, belongs to an important class of citrus flavonoids. Naringin acts on several neuroprotective antioxidants that function against oxidative neurotoxicity [27,28]. The protective roles of antioxidants are mediated through the modulation of TRPV1 [29]. Naringin treatment was found to mediate protective effects against erythrocytotoxicity and hippocampal oxidative neurotoxicity [27]. Naringin is also effective in treating obesity, diabetes, hypertension, and metabolic syndrome [30,31]. In addition, some studies have deemed naringin effective against pain. Naringin has been employed for the treatment of back pain [32] and osteoarthritis pain [33] in rats and humans and was found to exert neuroprotective effects through its anti-inflammatory, antioxidant, and anti-self-destruction activities [34].

The relationship between TRPV1 as a mediator of pain and naringin is yet unexplored. Here, we investigated the mechanism of interaction between naringin and TRPV1 at the molecular and cellular level. The purpose of this study was to confirm the potential of naringin as an ideal antagonist of TRPV1 and its applicability as a painkiller and antioxidant.

## 2. Materials and Methods

### 2.1. Materials

**Figure 2:**
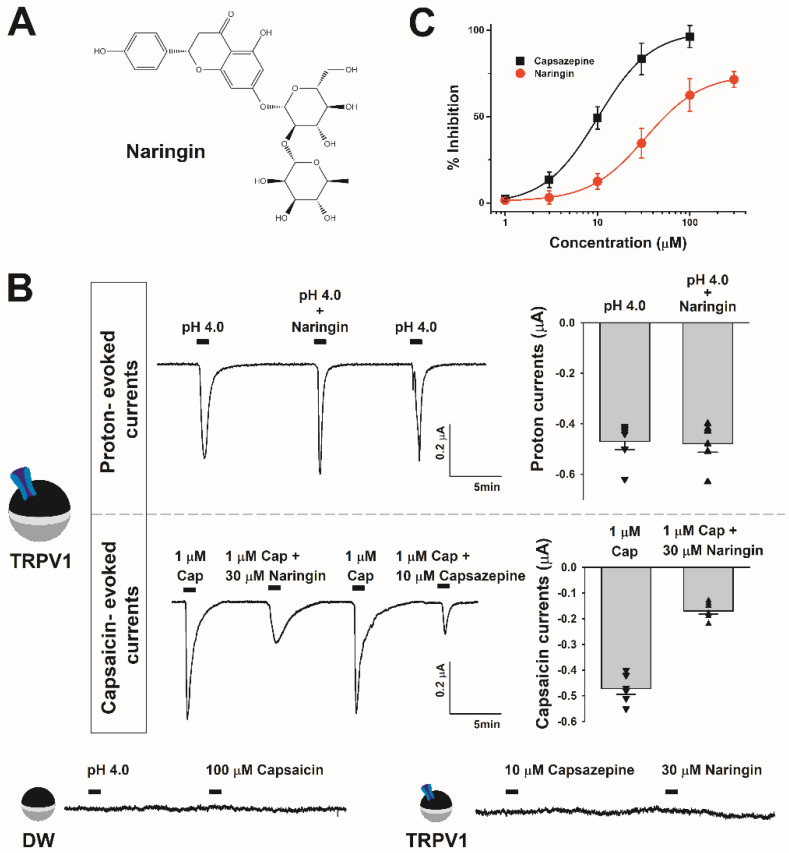
Selective inhibitory effect of capsaicin on TRPV1 and the chemical structure of naringin: (**A**) 2D structure of naringin. (**B**) Different inhibitory effects of naringin on proton-evoked currents and capsaicin-evoked currents. In the imaging of proton-evoked currents, naringin was used at a concentration of 30 μM. The TRPV1 oocyte image shows currents measured by injecting TRPV1 mRNA into oocytes. The distilled water-treated oocyte image shows currents measured by injecting tertiary distilled water into oocytes. Cap is capsaicin. Capsazepine is a published antagonist of TRPV1. The treatment was carried out at a pH of 4.0 using the ND96 buffer. (**C**) The histogram shows the degree of inhibition of TRPV1 inward current by naringin. The data are indicated as the mean ± S.E.M (*n* = 10–13 from six different frogs).

Rat TRPV1 cDNA (GenBank number: NM_031982) was obtained from OriGene (Rockville, MD, USA). Naringin was procured from Sigma-Aldrich (St. Louis, MO, USA). Figure 2A represents the chemical structure of naringin. Naringin was dissolved in dimethyl sulfoxide (DMSO) and further diluted in a bath solution to be used as a stock. The final DMSO concentration was less than 0.01%. *Xenopus laevis* was obtained from the Korean Xenopus Resource Center for Research (KXRCR000001), and other agents were purchased from Sigma-Aldrich.

### 2.2. In Vitro Transcription and Site-Directed Mutagenesis of TRPV1

Site-directed mutation of one or two amino acids was performed using sense and antisense primers and Pfu polymerase (QuikChange Site-Directed Mutagenesis kit; Agilent, Santa Clara, CA, USA). Point mutation was carried out by polymerase chain reaction (PCR) to increase the mutants of TRPV1. After removing the existing methylated cDNA using *Dpn*I, the PCR products were transformed into *Escherichia coli* strain DH5α. The cDNA of TRPV1 was linearized by *Xho*Ienzyme, which acted at the end of the multi-cloning site, and transcription was performed using SP6 polymerase and a transcription kit (mMESSAGE mMACHINE SP6 transcription kit; Thermo Fisher Scientific, Waltham, MA, USA). The RNA product was dissolved in 0.1% diethylpyrocarbonate (DEPC) water and stored at −80 °C.

### 2.3. Isolation of Xenopus Oocytes and TRPV1 mRNA Microinjection

To isolate oocytes, female *X. laevis* were put in a container filled with ice and left for about 2 h. Once the blood vessels contracted to a state similar to that observed during hibernation, the abdomen was incised. The ovaries were cut into small pieces and placed in Ca^2+^-free OR2 solution (82.5 mM sodium chloride (NaCl), 2.5 mM potassium chloride [KCl], 1 mM magnesium chloride (MgCl_2_), 5 mM HEPES, pH 7.4) containing 2 mg/mL collagenase. After 2 h of agitation to digest the follicle membrane, oocytes at stage V and VI were selected, isolated, and repeatedly washed. Selected oocytes were maintained in an ND96 incubation solution (96 mM NaCl, 2 mM KCl, 1.8 mM calcium chloride (CaCl_2_), 1 mM MgCl_2_, 5 mM HEPES, 2.5 mM sodium pyruvate, 50 µg/mL gentamycin, pH 7.4) at 16–18 °C. The solution containing oocytes was changed twice daily and stored in a shaking incubator. With the help of a microscope, the end of a 20 µm diameter glass capillary was broken; the glass capillary was filled with mineral oil, and air bubbles were discharged from the tip. The mRNA (40–50 ng) was injected into *Xenopus* oocytes using a nanoliter injector (Drummond Scientific, Broomall, PA, USA). Data collection started after 2 days of sufficient shaking in ND96 solution. Surgery, microinjection, and other handling of *X. laevis* were conducted following the manual protocol [35,36].

### 2.4. Voltage-Clamp Data Recording

Oocytes expressing mRNA during the incubation period were used to observe the potential interaction between the drug and ligand using TEVC (OC-725C; Warner Instruments, Hamden, CT, USA) and Digidata (1322A; Molecular Devices, Sunnyvale, CA, USA). TEVC has voltage and current electrodes that maintain a stable potential and deliver transmembrane potential and large currents. A voltage clamp amplifier transmits large currents of command potential, and Digidata converts the analog signal of the amplifier to enable reading. A computer converts output amplitude of voltage clamp amplifiers into input digital converter using pClamp 10 software (Axon Instruments, Union City, USA). Voltage and current electrodes were filled with 3 M KCl (0.3–0.7 MΩ), and the analysis was conducted at −70 mV holding potential. The oocytes were placed in a chamber and exposed to ND96 solution at a rate of 2 mL per minute. In electrophysiological experiments, the voltage ramp recording was performed at room temperature, and −100 to +80 mV potential was applied to study the current and voltage relationship. Inward peak traces expressed TRPV1, and voltage ramp traces were converted to a suitable value through Clampfit 9.0 (Molecular Devices, San Jose, CA, USA) [37,38].

### 2.5. Modeling and Molecular Docking

For molecular docking studies, the protein crystal structure of TRPV1 was obtained from RCSB Protein Data Bank (ID code: 3J5P). The three-dimensional structure of the ligand (naringin) was referenced in PubChem (CID code: 442428) and converted file type from “*.sdf” to “*.pdbqt”. Molecular docking studies were programmed using Autodock Tools of the Scripps Research Institute (version 4.2.6, La Jolla, CA, USA). Naringin and TRPV1 were docked with basic settings of the Autodock program, except for a few settings. We removed the water from the marcromolecule, added polarity and hydrogen, and charged the compute gasteiger [39]. We selected some models considering the intermolecular energy, inhibition constant, binding structures, and binding energy [40]. The selected complex of naringin and TRPV1 was analyzed using Ligplot (version 4.5.3, EMBL-EBI, Cambridge, UK) and Pymol (version 1.8.4.2, Schrödinger, New York, NY, USA). Ligplot showed binding activity between the ligand and protein. Pymol was used to measure the space between naringin and mutant amino acids of TRPV1.

### 2.6. Data Analysis

All data are represented as mean ± standard error of the mean (SEM). Concentration-dependent curves of capsazepine and naringin were fitted according to the Hill equation, y = V_min_ + (V_max_ − V_min_) × (X) [14] ^n^/(IC_50_^n^ + (X)^n^), where y is the serotonin-induced peak amplitude at various naringin concentrations, V_min_ and V_max_ are minimum and maximum values, respectively, (X) is naringin or capsaicin concentration, IC_50_ is the half-maximum inhibitory response concentration of naringin, and n is the interaction coefficient. All curves plotted through the Hill equation were represented using Origin Pro 9.0 (OriginLab, Northampton, MA, USA). The significance between the control and naringin treatment groups was determined according to the *p*-value, calculated using the Student’s *t*-test. The *p*-value was considered statistically significant when it was less than 0.05 [41].

## 3. Results

### 3.1. Concentration-Dependent and Reversible Effects of Naringin, Which Does Not Participate in Proton-Stimulated Activation and Only Inhibits Capsaicin-Stimulated TRPV1 Activation

We investigated the effects of naringin on capsaicin-induced inward peak current (*I*_cap_) and proton-induced inward peak current (*I*_H_) in TRPV1-expressing Xenopus oocytes using a two-electrode voltage clamp. We measured the regulating action of naringin on inward current at a membrane voltage potential of −80 mV. Figure 2A shows the chemical structure of naringin. In Xenopus oocytes injected with distilled water, no currents were observed following treatment with high concentration of capsaicin (100 μM, pH 2.5). No effect of protons and capsaicin was noted on Xenopus oocytes. As Xenopus oocytes have few endogenous membrane channels, they are suitable for receptor-ligand interaction studies. Xenopus oocytes injected with the TRPV1 mRNA were treated with 1 μM capsaicin, and inward peak currents were observed as shown in Figure 2B.

The current decreased following simultaneous treatment with 1 μM capsaicin and 30 μM naringin. We re-treated previously treated Xenopus oocytes with 1 μM capsaicin, and the same current was observed in the reversible manner. Therefore, naringin exhibited a reversible mode of action on TRPV1 in a concentration-dependent manner (Figure 2C). In addition, we compared the effect of naringin and capsazepine, an approved TRPV1 antagonist. The half-maximal inhibitory concentration (IC50) values of naringin and capsazepine were 33.3 ± 1.07 and 10.1 ± 0.02 μM, respectively, and the Hill coefficients were 1.43 ± 0.04 and 1.50 ± 0.01 (*n* = 7–12 from five different frogs), respectively.

### 3.2. Different Effects of Naringin on the Activity of Capsaicin and Protons (the Voltage–Current Relationship)

**Figure 3:**
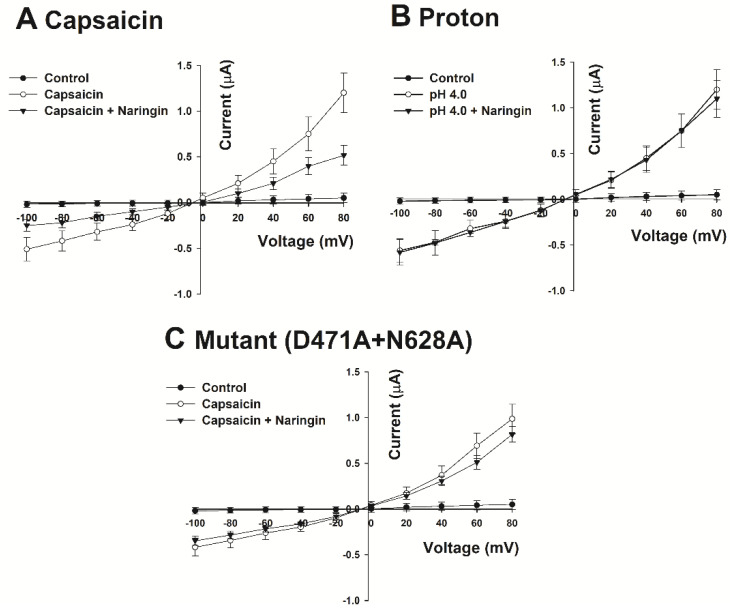
Differences in the current–voltage relationship according to different treatment conditions in TRPV1-expressing oocytes: (**A**) changes in capsaicin-induced current in response to planned voltage changes. The black circle (●) is the current measured in the oocytes injected with distilled water. White circles (○) are TRPV1-injected oocytes treated with 1 μM capsaicin. Black triangles (▼) show oocytes simultaneously treated with 1 μM capsaicin and 30 μM naringin. ●, ○, and ▼ in (**B**,**C**) also show the same trend. (**B**) Changes in proton-induced current in response to planned voltage changes. (**C**) Current–voltage relationship measured in oocytes expressing the double mutant D471A + N628A. These data are indicated as the mean ± S.E.M (*n* = 9–14 from six different frogs).

This experiment showed the effect of naringin according to the voltage change using the ramp protocol of TEVC. Each line in Figure 3 shows the current response in the oocyte membrane following exposure of eggs to increasing voltage (from −100 to +80 mV) for 1 s. First, the control line indicates the current measured only by voltage change in TRPV1-expressing oocytes. Considering that the current was maintained at 0 μA in the negative region from −100 to +80 mV when the resting potential was −90 to −50 mV, it could be confirmed that the current was maintained close to 0 μA even in the positive region where the voltage change was relatively large. The activation of TRPV1 was evident with voltage alone. However, following treatment, capsaicin could bind to TRPV1 and induce both capsaicin-stimulated inward currents in the negative region and capsaicin-stimulated outward currents in the positive region. In the presence of both naringin and capsaicin, the current induced by capsaicin was suppressed in all voltage areas, and the reversal potential remained unchanged. We performed this experiment in wild-type and double mutants and reported equal measures in the control and capsaicin-treated lines. However, the effect of the co-treatment with naringin and capsaicin was similar to that of capsaicin treatment alone. This result indicates that D471 and N628 of TRPV1 were only involved in the binding to naringin but not capsaicin.

### 3.3. Modeling and Docking of Naringin and TRPV1

**Figure 4:**
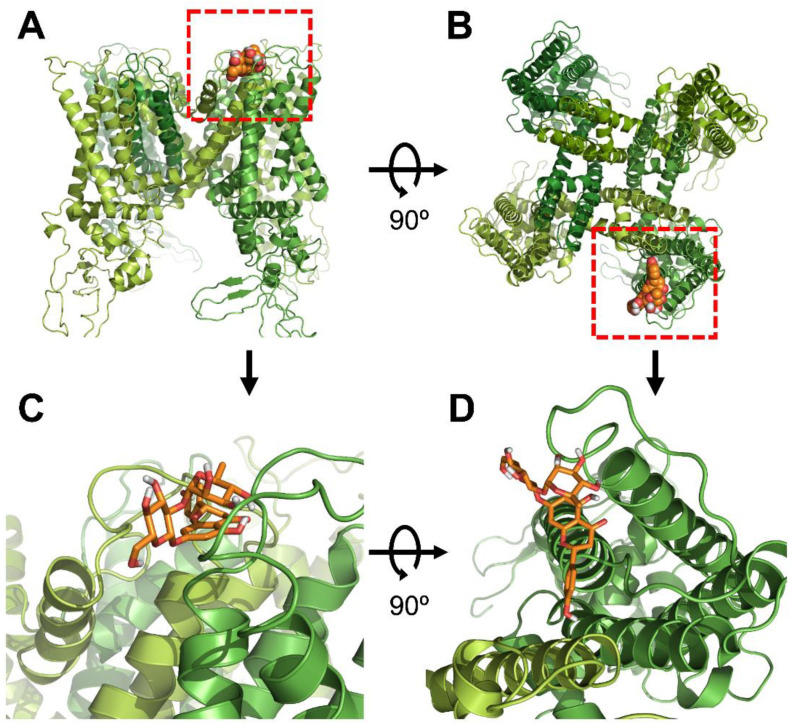
Molecular docking modeling of naringin to TRPV1: (**A**) side views of the docked naringin in complex with TRPV1. (**B**) Top views of the docking model. (**C**) An enlarged view of the red dotted line in Figure 4A. (**D**) An enlarged view of the red dotted line in Figure 4B.

We derived the expected binding sites of TRPV1 and naringin through docking. This position was different from the binding position of capsaicin. Figure 4 shows the results for wild-type TRPV1 (ID code 3J5P), which has a known crystal structure. Through computational simulation, the most stable binding site between naringin and TRPV1 was predicted to be near the S1–S2 loop located at the extracellular side.

**Figure 5:**
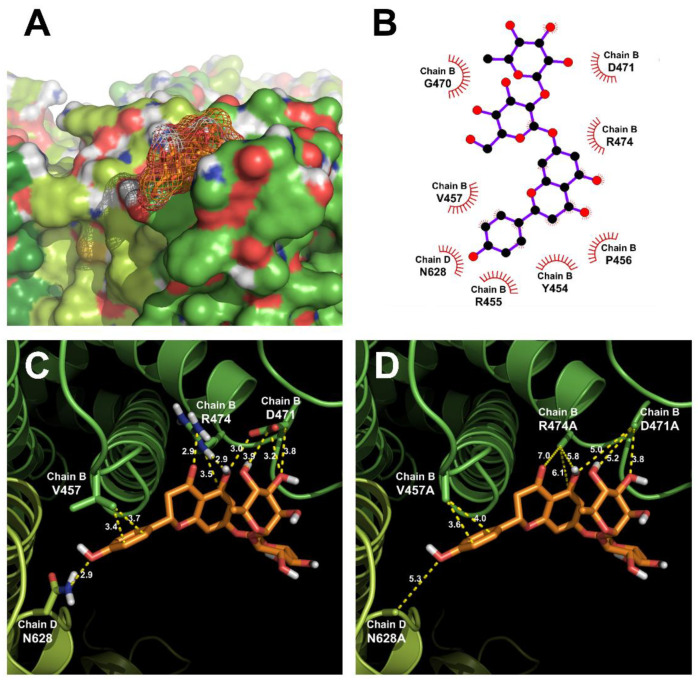
The binding pocket and docking results of naringin and TRPV1: (**A**) the binding pocket in the TRPV1 region of the extracellular domain membrane pocket side. (**B**) Two-dimensional schematic presentation of the predicted binding mode of naringin in the ligand-binding pocket. The ligands and important residues are shown. (**C**,**D**) Computational simulated binding interaction of ligand and residues in wild-type and mutants. The replaced mutants showed changes in interaction activities at varying degrees. (**C**) Interaction between naringin and wild-type TRPV1. (**D**) Interaction between naringin and mutant-type TRPV1.

As per the docking result shown in Figure 5A, the binding energy was −2.58 kcal/mol, intermolecular energy was −6.75 kcal/mol, internal energy was −6 kcal/mol, torsional energy was 4.18 kcal/mol, unbound extended energy was −6 kcal/mol, refRMS was 58.11, and Ki was 12.93 mM. Specific amino acid residues, such as D471 and N628, are shown in Figure 5B. Based on these results, we conclude that D471 and N628 were involved in naringin binding owing to the point mutation of predicted residues and expression in frog eggs. We confirmed these observations using TEVC. In addition, the change in the distance between atoms was measured using Pymol when the predicted bonding amino acid residue was mutated with alanine. Therefore, the interatomic distances of D471 changed from 3.0, 3.9, 3.2, and 3.8 Å to 5.0, 5.2, and 3.8 Å, respectively. N628 decreased the bond ability and increased the interatomic distance from 2.9 to 5.3 Å (Figure 5).

### 3.4. Cross-Checking by Electrophysiology after Mutant Production

**Figure 6:**
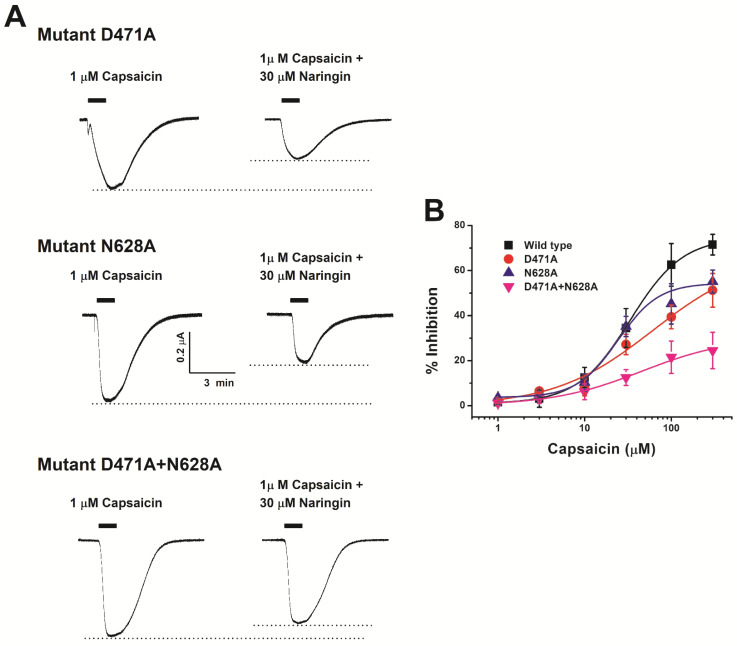
Comparison of the effect of naringin on mutant TRPV1: (**A**) D471A mutant TRPV1 was co-treated with naringin (30 μM) and capsaicin (1 μM). D471A mutant group was injected only with D471A TRPV1 mRNA into oocytes. N628A mutant TRPV1 was co-treated with naringin (30 μM) and capsaicin (1 μM). N628A mutant group was only injected with N628A TRPV1 mRNA into oocytes. D471A + N628A mutant group was injected with both D471A and N628A TRPV1 mRNAs into oocytes. (**B**) Concentration-response curves for the effect of naringin on oocytes expressing mutants. The percent inhibition of *I_cap_* on each mutant was normalized based on the peak inward current induced by capsaicin and that of the peak inward current elicited by capsaicin plus naringin. Each point showed the mean ± S.E.M. (*n* = 9–14 from six different frogs). Additional half inhibitory concentration, Hill coefficient, and Imax values are described in Results.

Based on the above results, a mutant-type TRPV1 wherein the amino acid corresponding to the expected binding site was point-mutated with alanine was produced. TEVC was used to investigate whether there was any change in inhibition due to naringin. We first confirmed that the mutants operated like wild-type TRPV1, except for their binding with naringin. As shown in Figure 6, each mutant TRPV1 showed a capsaicin-induced current similar to that of wild-type TRPV1. Inhibition was reduced in D471A and N628A as compared to that in wild-type TRPV1. After fitting with the Hill equation (Table 1), the V_max_ was calculated to be 66.6 for mutant D471A and 54.4 for mutant N628A. In comparison with the V_max_ of wild-type TRPV1 (27.2%), mutant TRPV1 showed decreased interaction with naringin (10.8%). Therefore, we investigated whether there occurred any change in the effect of naringin by constructing a mutant with two amino acid mutations. The V_max_ of wild-type TRPV1 was 74.7, and that of D471A + N628A double mutant was 30.4. In comparison with the wild-type TRPV1, the double mutant had a 60% decrease in V_max_. The interactions between naringin and TRPV1 at aspartate, the 471st amino acid residue, and asparagine, the 628th amino acid residue, were closely related but not involved in the normal operation of TRPV1.

## 4. Discussion

Neuropathic pain from oxidation is a mechanism that lowers the quality of life. There is no efficient painkiller that could curb pain in humans. Investigating the pain-suppressing mechanism of TRPV1, rather than studying conventional mechanisms, is considered reliable for the development of new painkillers [18,19,22]. Therefore, we screened natural substances that have been reported to exhibit neuronal protective effects and that are likely to interact with TRPV1. In our previous study [42], the mechanism of the interaction between TRPV1 and gomisin A was elucidated. However, some parts of gomisin A were unsuitable as analgesic candidates. In this study, we found that naringin selectively inhibited capsaicin-evoked activity but not proton-evoked activity. We elucidated the mechanism by which naringin could regulate TRPV1 and investigated its binding site. Our findings at the cellular and molecular levels revealed the basic yet useful information for comparison with other drugs or to develop new drugs in future studies.

The IC_50_ value of capsazepine, a TRPV1 representative antagonist, was 10.1 ± 0.02 μM, while gomisin A from our previous study had an IC_50_ value of 62.1 μM. Naringin investigated in the present study showed an IC_50_ value of 33.3 μM. Thus, naringin showed higher potency and efficiency than gomisin A. While many of the binding sites of conventionally identified TRPV1 were located in the S5–S6 loop, gomisin A and naringin interacted with the previously unknown S1–S2 loop; thus, the binding sites could also exist in the S1–S2 loop. TRPV1 seems to have interactive amino acid residues in almost every location and acts as an integrator that senses various stimuli both outside and inside the cell.

A previous study showed that the inhibition of TRPV1 was effective in neuropathic pain model rats. Two-stage experiments revealed the effective inhibition of TRPV1 in the management of osteoarthritis knee pain. In addition, other investigations on antagonists are currently underway.

Capsaicin is a representative drug agonist of TRPV1. High interaction between TRPV1 and capsaicin activated the mechanism of desensitization in the neuron, which does not generate capsaicin-stimulated currents because of the reduction in the number of TRPV1 molecules in membranes. The analgesic effect by antagonism of TRPV1 is a strategy to reduce the electrical signal through the inhibition of the flow of ions via TRPV1 but not through the decrease in the number of TRPV1 receptors in the membrane. Capsaicin is the only FDA-approved agonist, as the development of antagonists is challenging.

One of the main reasons underlying the failure of first-generation antagonists is the high rate of fever developed by recipients. First-generation non-selective antagonists are involved in regulation by hydrogen or heat as well as the inactivation of capsaicin. Thus, the drugs involved in the regulation of capsaicin, but not hydrogen or heat, were chosen as second-generation selective antagonists. Drugs that inhibit capsaicin-induced inward-current but are not involved in hydrogen-induced activity were suggested to be ideal TRPV1 antagonists for treating pain. The method currently used in our study was to express TRPV1 in *Xenopus* oocytes and to measure the interaction mechanism between TRPV1 and naringin using TEVC. As *Xenopus* oocytes are damaged by heat when the external environment is above 18 °C, their activity above 40 °C was not measured in the present study. However, our findings show that naringin was not involved in the proton activity and only inhibited *I*_cap_. The interaction position of protons was between S5 and S6, while that of naringin was between S1 and S2 (Figure 1A). The use of these different coupling positions is one of the key reasons why naringin selectively inhibited *I*_cap_.

As mentioned above, several studies have investigated the neuroprotective and pain inhibitory effect of naringin, which exhibits anti-apoptosis, antioxidant, and anti-inflammatory properties. It has been known that oxidative stress is reduced through inhibition 8of glutathione (GSH), malondialdehyde (MDA), and nitrite in neuronal cells and the hippocampus of mice fed with naringin. Mitochondrial oxidative damage is reduced by the activation of superoxide dismutase (SOD), catalase (CAT), and glutathione-S-transferase (GST) (Figure 1C). As naringin effectively passes through the blood–brain barrier (BBB), it protects neuronal damage through antioxidants in the central nervous system as well as the peripheral nervous system [43]. In humans, the terminal elimination half-life of naringin was at least 1.13 ± 0.63 h, with a maximum value of 3.86 ± 2.02 h. Toxicity evaluation [44] after oral administration of naringin (1250 mg/kg/day for 6 months) to Sprague–Dawley rats showed no clinical signs and changes attributable to lesions and pathology. In particular, some studies reported that naringin was effective for osteoarthritis pain [33] and back pain [32] and that the antagonist of TRPV1 was effective for osteoarthritis pain [45] or neuropathy pain [46]. Capsazepine, an antagonist of TRPV1, exerted protective effects against psychiatric disorders such as anxiety and depression. Several reports have revealed the protective effects of naringin on mental and neurodegenerative disorders. However, there is a dearth of studies on the interaction between TRPV1 and naringin.

In summary, we demonstrate that naringin acted in a concentration-dependent and voltage-insensitive manner, reversibly inhibited the interaction with TRPV1 at the cellular and molecular level, and had an IC_50_ value of 33.33 μM. Naringin inhibited only *I*_cap_ without affecting *I*_H_. We cross-checked the results from TEVC and docking experiments and found that D471 and N628 of TRPV1 were involved in the binding with naringin (Figure 6B).

## 5. Conclusions

In conclusion, our findings will bridge the gap between the pain reduction effect of naringin and the role of TRPV1 antagonists in the development of new analgesics. Moreover, the characteristic of naringin, which inhibits only *I*_cap_ without being involved in *I*_H_, is similar to that of the model-selective TRPV1 antagonist (second-generation model) that is currently the most ideal model for the development of analgesics. Therefore, future studies should investigate whether osteoarthritis pain and neuropathic pain are reduced through the inhibition of TRPV1 activity caused by naringin at a preclinical or clinical stage. Furthermore, it seems necessary to study how the interaction between TRPV1 and naringin affects mental disorders and neurodegenerative diseases. These follow-up studies will take us one step closer in the development of antioxidants that could be effective in neuropathic pain management.

## Figures and Tables

**Table 1:** Inhibitory effect of naringin on wild-type and mutant TRPV1 receptors.

Model	Hill Equation		
Equation	y = V_min_ + (V_max_ − V_min_) × x^*n*^/(k^*n*^ x^*n*^)		
		Value	Standard Error
Wild type	V_min_	0.990926	0.127888
	V_max_	74.67297	0.672273
	k	33.32861	1.06873
	*n*	1.42207	0.045205
Mutant D471A	V_min_	−0.04689	4.090147
	V_max_	66.63634	33.60697
	k	60.56565	86.38281
	*n*	0.770134	0.400584
Mutant N628A	V_min_	3.648402	0.760874
	V_max_	54.39192	3.48829
	k	24.17166	3.622099
	*n*	1.844236	0.384702
Mutant D471A+N628A	V_min_	0.10516	1.05177
	V_max_	30.43531	6.377348
	k	45.48037	24.45853
	*n*	0.841358	0.24784

The values represent mean ± S.E.M (*n* = 9–14 from six different frogs). Currents were elicited at a holding potential of −70 mV. IC_50_ (μM), Hill coefficient (*n*), and V_max_ (%) were determined as described in Materials and Methods.

## Data Availability

Data is contained within the article.

## References

[1] Haraoui N., Allem R., Chaouche T.M., Belouazni A. (2020). In-vitro antioxidant and antimicrobial activities of some varieties citrus grown in Algeria. Adv. Tradit. Med..

[2] Bezverkhniaia E.A., Ermilova E.V., Kadyrova T.V., Krasnov E.A., Brazovskii K.S., Ponkratova A.O., Luzhanin V.G., Belousov M.V. (2021). Phytochemistry, ethnopharmacology and pharmacology of the genus Empetrum: A review. Adv. Tradit. Med..

[3] Lee H., Ahn S., Ann J., Ha H., Yoo Y.D., Kim Y.H., Hwang J.-Y., Hur K.-H., Jang C.-G., Pearce L.V. (2019). Discovery of dual-acting opioid ligand and TRPV1 antagonists as novel therapeutic agents for pain. Eur. J. Med. Chem..

[4] Loeser J.D., Melzack R. (1999). Pain: An overview. Lancet.

[5] Nicholson B. (2006). Differential diagnosis: Nociceptive and neuropathic pain. Am. J. Manag. Care.

[6] Campbell J.N., Meyer R.A. (2006). Mechanisms of neuropathic pain. Neuron.

[7] Colloca L., Ludman T., Bouhassira D., Baron R., Dickenson A.H., Yarnitsky D., Freeman R., Truini A., Attal N., Finnerup N.B. (2017). Neuropathic pain. Nat. Rev. Dis. Primers.

[8] Baliki M.N., Apkarian A.V. (2015). Nociception, pain, negative moods, and behavior selection. Neuron.

[9] Julius D., Basbaum A.I. (2001). Molecular mechanisms of nociception. Nature.

[10] Wang V.C., Mullally W.J. (2020). Pain neurology. Am. J. Med..

[11] Cohen S.P., Mao J. (2014). Neuropathic pain: Mechanisms and their clinical implications. BMJ.

[12] Baron R., Binder A., Wasner G. (2010). Neuropathic pain: Diagnosis, pathophysiological mechanisms, and treatment. Lancet Neurol..

[13] Kaneko Y., Szallasi A. (2014). Transient receptor potential (TRP) channels: A clinical perspective. Br. J. Pharmacol..

[14] Samanta A., Hughes T.E., Moiseenkova-Bell V.Y. (2018). Transient receptor potential (TRP) channels. Membr. Protein Complexes Struct. Funct..

[15] Cao E., Liao M., Cheng Y., Julius D. (2013). TRPV1 structures in distinct conformations reveal activation mechanisms. Nature.

[16] Aghazadeh Tabrizi M., Baraldi P.G., Baraldi S., Gessi S., Merighi S., Borea P.A. (2017). Medicinal chemistry, pharmacology, and clinical implications of TRPV1 receptor antagonists. Med. Res. Rev..

[17] Zhang M., Ruwe D., Saffari R., Kravchenko M., Zhang W. (2020). Effects of TRPV1 activation by capsaicin and endogenous n-arachidonoyl taurine on synaptic transmission in the prefrontal cortex. Front. Neurosci..

[18] Trevisani M., Gatti R. (2013). TRPV1 antagonists as analgesic agents. Open Pain J..

[19] Garami A., Shimansky Y.P., Rumbus Z., Vizin R.C., Farkas N., Hegyi J., Szakacs Z., Solymar M., Csenkey A., Chiche D.A. (2020). Hyperthermia induced by transient receptor potential vanilloid-1 (TRPV1) antagonists in human clinical trials: Insights from mathematical modeling and meta-analysis. Pharmacol. Ther..

[20] Duarte Y., Cáceres J., Sepúlveda R.V., Arriagada D., Olivares P., Díaz-Franulic I., Stehberg J., González-Nilo F. (2020). Novel TRPV1 channel agonists with faster and more potent analgesic properties than capsaicin. Front. Pharmacol..

[21] Bölcskei K., Helyes Z., Szabó Á., Sándor K., Elekes K., Németh J., Almási R., Pintér E., Pethő G., Szolcsányi J. (2005). Investigation of the role of TRPV1 receptors in acute and chronic nociceptive processes using gene-deficient mice. Pain.

[22] Morales-Lázaro S.L., Simon S.A., Rosenbaum T. (2013). The role of endogenous molecules in modulating pain through transient receptor potential vanilloid 1 (TRPV1). J. Physiol..

[23] Sondermann J.R., Barry A.M., Jahn O., Michel N., Abdelaziz R., Kügler S., Gomez-Varela D., Schmidt M. (2019). Vti1b promotes TRPV1 sensitization during inflammatory pain. Pain.

[24] Green D.P., Ruparel S., Roman L., Henry M.A., Hargreaves K.M. (2013). Role of endogenous TRPV1 agonists in a postburn pain model of partial-thickness injury. Pain.

[25] Honore P., Chandran P., Hernandez G., Gauvin D.M., Mikusa J.P., Zhong C., Joshi S.K., Ghilardi J.R., Sevcik M.A., Fryer R.M. (2009). Repeated dosing of ABT-102, a potent and selective TRPV1 antagonist, enhances TRPV1-mediated analgesic activity in rodents, but attenuates antagonist-induced hyperthermia. Pain.

[26] Arsenault P., Chiche D., Brown W., Miller J., Treister R., Leff R., Walker P., Katz N. (2018). NEO6860, modality-selective TRPV1 antagonist: A randomized, controlled, proof-of-concept trial in patients with osteoarthritis knee pain. Pain Rep..

[27] Akamo A.J., Akinloye D.I., Ugbaja R.N., Adeleye O.O., Dosumu O.A., Eteng O.E., Antiya M.C., Amah G., Ajayi O.A., Faseun S.O. (2021). Naringin prevents cyclophosphamide-induced erythrocytotoxicity in rats by abrogating oxidative stress. Toxicol. Rep..

[28] Karthikeyan A., Kim H.H., Preethi V., Moniruzzaman M., Lee K.H., Kalaiselvi S., Kim G.S., Min T. (2021). Assessment of anti-inflammatory and antioxidant effects of citrus unshiu peel (CUP) flavonoids on LPS-stimulated RAW 264.7 cells. Plants.

[29] Düzova H., Nazıroğlu M., Çiğ B., Gürbüz P., Akatlı A.N. (2021). Noopept Attenuates diabetes-mediated neuropathic pain and oxidative hippocampal neurotoxicity via inhibition of trpv1 channel in rats. Mol. Neurobiol..

[30] Bell R.F., Borzan J., Kalso E., Simonnet G. (2012). Food, pain, and drugs: Does it matter what pain patients eat?. Pain.

[31] Alam M.A., Subhan N., Rahman M.M., Uddin S.J., Reza H.M., Sarker S.D. (2014). Effect of citrus flavonoids, naringin and naringenin, on metabolic syndrome and their mechanisms of action. Adv. Nutr..

[32] Li N., Whitaker C., Xu Z., Heggeness M., Yang S.-Y. (2016). Therapeutic effects of naringin on degenerative human nucleus pulposus cells for discogenic low back pain. Spine J..

[33] Xu Q., Zhang Z.-F., Sun W.-X. (2017). Effect of naringin on monosodium iodoacetate-induced osteoarthritis pain in rats. Med. Sci. Monit..

[34] Ahmed S., Khan H., Aschner M., Hasan M.M., Hassan S.T. (2019). Therapeutic potential of naringin in neurological disorders. Food Chem. Toxicol..

[35] Maldifassi M.C., Wongsamitkul N., Baur R., Sigel E. (2016). Xenopus oocytes: Optimized methods for microinjection, removal of follicular cell layers, and fast solution changes in electrophysiological experiments. J. Vis. Exp. JoVE.

[36] Aguero T., Newman K., King M.L. (2018). Microinjection of Xenopus oocytes. Cold Spring Harb. Protoc..

[37] Guan B., Chen X., Zhang H. (2013). Two-electrode voltage clamp. Ion Channels.

[38] Schreibmayer W., Lester H.A., Dascal N. (1994). Voltage clamping of Xenopus laevis oocytes utilizing agarose-cushion electrodes. Pflügers Arch..

[39] Eom S., Jung W., Lee J., Yeom H.D., Lee S., Kim C., Park H.-D., Lee J.H. (2021). Differential regulation of human serotonin receptor type 3A by chanoclavine and ergonovine. Molecules.

[40] Basak S., Gicheru Y., Kapoor A., Mayer M.L., Filizola M., Chakrapani S. (2019). Molecular mechanism of setron-mediated inhibition of full-length 5-HT 3A receptor. Nat. Commun..

[41] Lee S., Jung W., Eom S., Yeom H.D., Park H.-D., Lee J.H. (2021). Molecular regulation of betulinic acid on α3β4 nicotinic acetylcholine receptors. Molecules.

[42] Lee S.B., Noh S., Yeom H.D., Jo H., Eom S., Kim Y.S., Nam S., Bae H., Lee J.-H. (2017). A molecular basis for the inhibition of transient receptor potential vanilloid type 1 by Gomisin, A. Evid.-Based Complement. Altern. Med..

[43] Tsai T.-H. (2002). Determination of naringin in rat blood, brain, liver, and bile using microdialysis and its interaction with cyclosporin a, a p-glycoprotein modulator. J. Agric. Food Chem..

[44] Li P., Wang S., Guan X., Cen X., Hu C., Peng W., Wang Y., Su W. (2014). Six months chronic toxicological evaluation of naringin in Sprague–Dawley rats. Food Chem. Toxicol..

[45] Quiding H., Jonzon B., Svensson O., Webster L., Reimfelt A., Karin A., Karlsten R., Segerdahl M. (2013). TRPV1 antagonistic analgesic effect: A randomized study of AZD1386 in pain after third molar extraction. Pain.

[46] Schwartz E.S., La J.H., Scheff N.N., Davis B.M., Albers K.M., Gebhart G.F. (2013). TRPV1 and TRPA1 antagonists prevent the transition of acute to chronic inflammation and pain in chronic pancreatitis. J. Neurosci..

